# Design of a Bio-Inspired Gait Phase Decoder Based on Temporal Convolution Network Architecture With Contralateral Surface Electromyography Toward Hip Prosthesis Control

**DOI:** 10.3389/fnbot.2022.791169

**Published:** 2022-05-09

**Authors:** Yixi Chen, Xinwei Li, Hao Su, Dingguo Zhang, Hongliu Yu

**Affiliations:** ^1^School of Health Science and Engineering, University of Shanghai for Science and Technology, Shanghai, China; ^2^Shanghai Engineering Research Center of Assistive Devices, Shanghai, China; ^3^School of Mechanical Engineering, Institute of Robotics, Shanghai Jiao Tong University, Shanghai, China; ^4^Laboratory of Biomechatronics and Intelligent Robotics (BIRO), Department of Mechanical and Aerospace Engineering, North Carolina State University, Raleigh, NC, United States; ^5^Department of Electronic and Electrical Engineering, University of Bath, Bath, United Kingdom

**Keywords:** gait phase, gait coordination, lower limb prosthesis, sensor fusion, sEMG, temporal convolution network, detrended cross-correlation analysis (DCCA), hip disarticulation

## Abstract

Inter-leg coordination is of great importance to guarantee the safety of the prostheses wearers, especially for the subjects at high amputation levels. The mainstream of current controllers for lower-limb prostheses is based on the next motion state estimation by the past motion signals at the prosthetic side, which lacks immediate responses and increases falling risks. A bio-inspired gait pattern generation architecture was proposed to provide a possible solution to the bilateral coordination issue. The artificial movement pattern generator (MPG) based on the temporal convolution network, fusing with the motion intention decoded from the surface electromyography (sEMG) measured at the impaired leg and the motion status from the kinematic modality of the prosthetic leg, can predict four sub gait phases. Experiment results suggested that the gait phase decoder exhibited a relatively high intra-subject consistency in the gait phase inference, adapted to various walking speeds with mean decoding accuracy ranging from 89.27 to 91.16% across subjects, and achieved an accuracy of 90.30% in estimating the gait phase of the prosthetic leg in the hip disarticulation amputee at the self-selected pace. With the proof of concept and the offline experiment results, the proposed architecture improves the walking coordination with prostheses for the amputees at hip level amputation.

## 1. Introduction

High-level amputation like hip disarticulation requires the utilization of an intelligent prosthesis to restore the amputee's natural gait. One way to achieve this goal is to employ a phase-based controller (Tucker et al., 2015). Currently, the powered prosthetic leg usually determines the gait phase merely according to its motion parameters (Wang et al., 2013; Chen et al., 2015; Gao et al., 2019; Fluit et al., 2020). The lack of immediate contralateral information will lead to coordination errors between the healthy leg and the prosthetic leg and induce the irregular motor relearning process which may cause the additional trajectory displacement of the body's center of gravity (Askew et al., 2019). As a consequence of the increasing conscious efforts and physical workload, the abnormal gait pattern may limit the wearer's locomotion ability and result in greater energy expenditure during plane walking. Most important of all, the accumulated coordination errors will induce a higher falling risk. Thus, it is necessary for the intelligent prosthesis to determine the gait phase based on bilateral information.

However, there are only limited researches that focus on decoding the fused bilateral sensory feedback to predict the rhythmic gait phase during cyclic bipedal walking. Echo control is one implementation of such rhythm generator to replay the joint motion trajectory of the sound leg at the prosthetic side with an appropriate phase delay. With the contralateral motion information as the reference input and the prosthetic leg kinematic status as the real-time control feedback, it is easier to achieve coordination response characteristics. An instance of a gait phase decoder based on this echo paradigm can be found in Wang et al. (2013), but this solution is indirect, as the motion intention is estimated from the delayed motion. Besides, the decoder's dynamic performances are not fully estimated in case the walking speed changes.

Essentially, human gait is the consequence of complex motor activities under the high-level coordination between the trunk and the lower limbs. Movement Pattern Generator (MPG) is proposed by scientists to explain the emerging coordination mechanism from the bottom up. One definition describes the gait MPG as the neural circuits localized in the spinal cord with the capability to produce a periodic rhythmic pattern during walking (Dzeladini et al., 2017). A typical gait MPG architecture involves the sensory feedback of gait state monitoring and the information fusion mechanics to generate the movement to accomplish certain gait tasks.

An artificial MPG architecture may offer a solution to overcome the shortcomings of the current gait phase controller, and it provides an inspiring insight in 2-folds. On the one hand, MPG can fuse the sensory feedback of bilateral information at the spinal cord level which provides a mid-level control strategy. The amputee-prosthesis coupling system is a typical biological-physical-cyber system, the motion intention can be decoded from the physiological signals captured during the activity pattern of human motors and it can be mapped to the gait sub-phase with a specific physical meaning. The surface electromyography (sEMG) is convenient to access (Xiong et al., 2021), provides abundant neural command information (Farina et al., 2014), and precedes the motion of the actuated limb (Jiang et al., 2012; Copaci et al., 2018). These advantages make it a priority among other biological modalities related to motion. Moon et al. (2019) reported a faster detection of 27.1 ms on average when fusing sEMG sensors to control the knee exoskeleton. Their design can guarantee an immediate response when the gait speeds change and meet the basic daily demands of the amputee. Besides, if motion intention is decoded from the healthy leg of the amputee, it may provide a better choice to overcome the shortcomings of the poor signal quality when the sEMG was captured from the residual limb (Fleming et al., 2021). Moreover, the sEMG is especially suitable for monitoring muscle activity patterns during walking, this can provide an additional benefit in the gait restoration assessment at the same time.

On the other hand, an MPG helps to stabilize gait against perturbations (Duysens and Forner-Cordero, 2018). This can be critical to enhancing the gait coordination robustness under the speed variation. Recently, with the multiple-domain success, the neural network modeling approach has highlighted its robust capability of approximately realizing any continuous mapping, including both linear and nonlinear representation (Funahashi, 1989). The mapping from sEMG to the gait phase is of high nonlinearity, so neural networks become the candidate to tackle the sEMG-based gait phase decoding problem. Furthermore, deep learning techniques are introduced to improve the performance of the artificial neural network. Morbidoni et al. (2019) utilized a deep network with 2–5 hidden layers to solve the stance/swing phases classification in natural walking scenarios and reported an average binary classification accuracy of 94.9% for learned subjects and 93.4% for unlearned ones. The current existing deep learning approaches are robust enough to overcome the weakness of sEMG, thus, achieving higher accuracy. Additionally, a more subtle granularity of gait phase recognition requires a more sophisticated approach. Luo et al. (2020) designed an sEMG-based real-time gait phase recognition system using a classifier combining long-short term memory (LSTM) with multilayer perceptron to make a four gait-phase-classification prediction. Their experiment results of walking on flat terrain at 5 km/h and 3 km/h achieved an average classification accuracy of 94.10 and 87.25%, respectively. It should be noted, LSTM introduces the memory mechanism to enhance the network's ability, which allows the network to utilize both the current status and the previous information. Since walking is a continuous process, the relationship between different sub-phases is embedded within the time-series order. Thus, the gait phase decoding can be restated as a sequence modeling problem, and this makes gait phase recognition different from the typical classification tasks (where the order does not matter) when selecting the neural network.

To sum up, in order to provide coordinated gait assistance, the phase-based controller of the intelligent prosthetic leg should be able to decode the gait phase not only according to its motion parameters but also based on the physiological information from the healthy legs. In this study, preliminary experiments on six healthy volunteers and one hip disarticulation amputee were conducted to explore the rationality of this bio-inspired architecture. To be more specific, we first checked the availability of the neural network approach to decode the gait phase from fused modalities of the dominant leg during plane walking under different speeds, and then tested the bilateral muscle activities correlation as well as examining whether there was a significant difference between decoding results using modalities from the ipsilateral and the contralateral. Finally, we validated the design philosophy with offline data obtained from one unilateral hip disarticulation amputee. The key contributions of the present research are: 1) Provide a novel proof-in-conception prosthesis myocontrol approach with a focus on hip disarticulation amputees; 2) Investigate the performance of gait phase decoder fusing bilateral neuromechanical signals.

## 2. Methods

**Figure 1:**
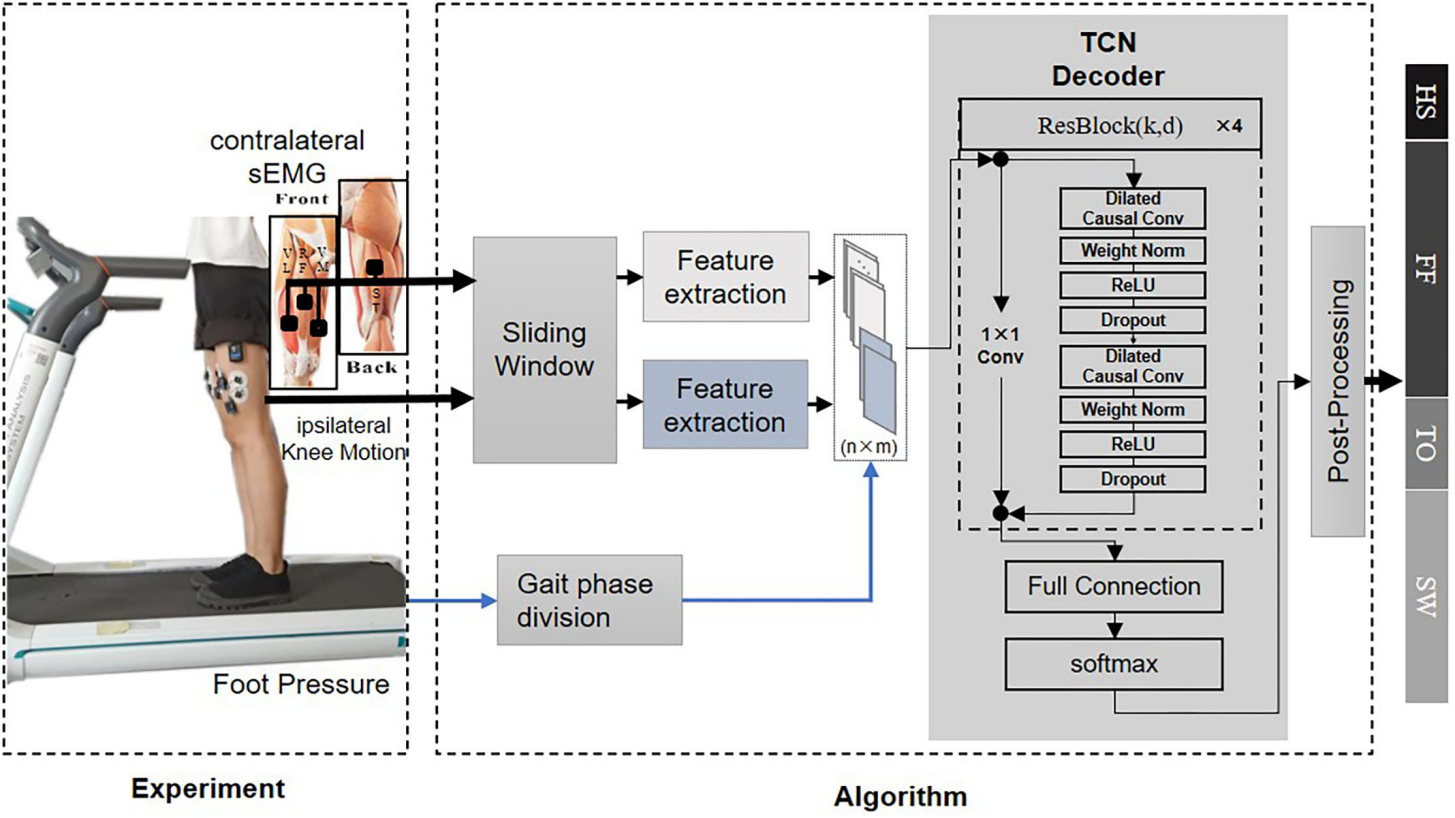
Overview of the data acquisition scenario and architecture of the gait phase decoder in the offline mode. The decoder fuses simultaneous sensory feedback from the contralateral surface electromyography (sEMG) and the ipsilateral knee motion to generate an inference out of four-gait-phase categories which include HS (heel strike), FF (flat feet), TO (toe off), and SW (swing). The foot pressure is utilized for the labeling process. Front and rear view of four thigh muscles including Semitendinosus (ST), Rectus Femoris (RF), Vastus Medialis (VM), and Vastus Lateralis (VL) is inserted to exhibit the electrode placement. All the serially connected residual blocks (ResBlock for short) share the same structure, thus, only one block is unfolded for illustration.

The function of the proposed gait phase decoder, as well as a scene of the experiment, can be perceived in Figure 1. With the ability to classify the time sequence, the decoder makes an inference about the current gait phase state from four-gait-phase categories based on bilateral sensory feedback. Furthermore, we defined two metrics to evaluate the decoder's performance. Later, we described the experimental protocol and statistical techniques in detail for the algorithm evaluation.

### 2.1. Algorithm Design

#### 2.1.1. Preprocessing

**Figure 2:**
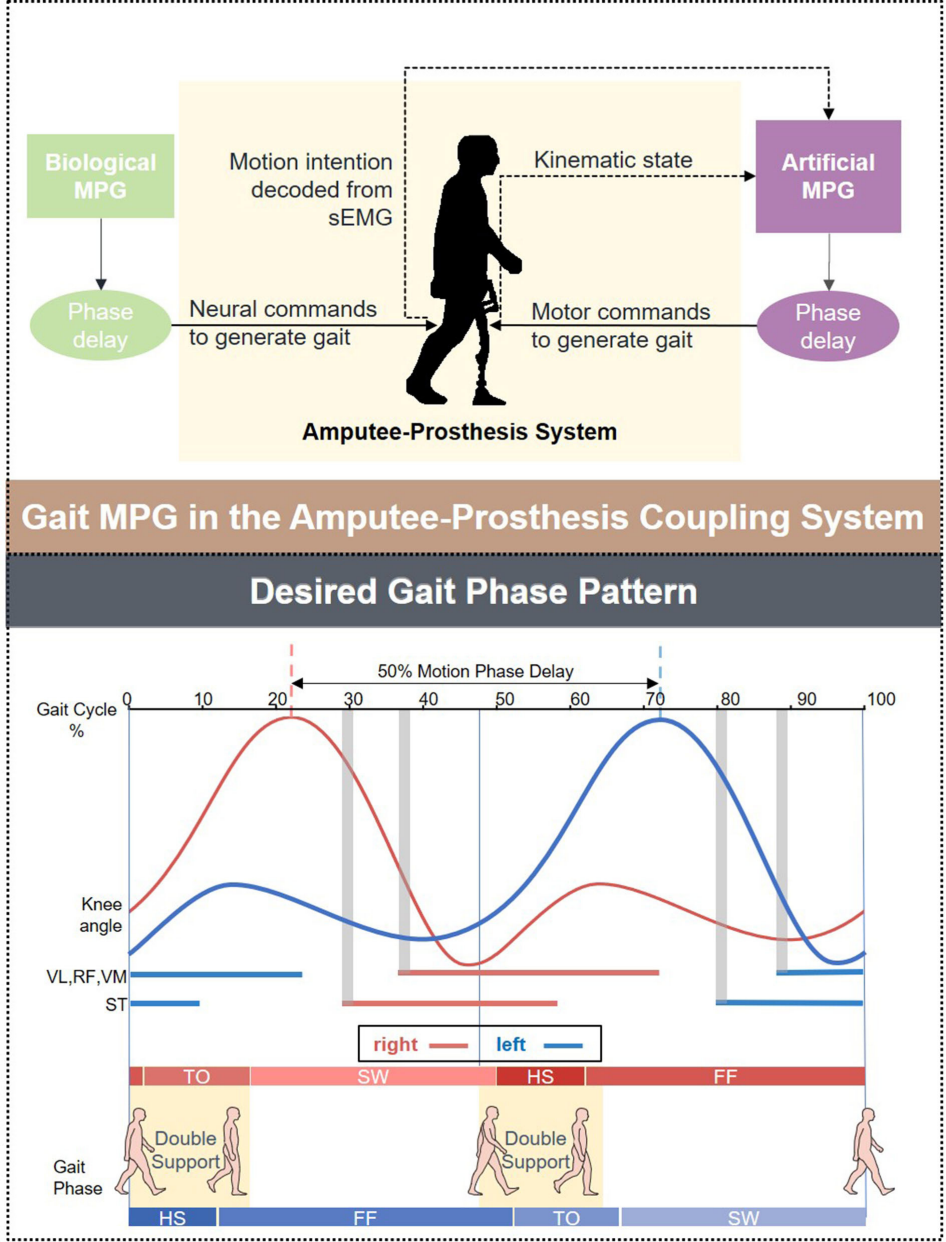
An illustration explains how the coupled movement pattern generator (MPG) architecture produces the desired gait pattern. The schema above shows the information flow between the prosthetic leg and the healthy leg, while a diagram below presents the bilateral knee angle curves and relevant muscles' onset patterns in one gait cycle. The interval between the dotted lines represents bilateral phase delay. The width of the gray rectangle stands for electromechanical delay (EMD).

Surface electromyography is the electrical potential captured in the skin during the muscle fibers contract and relax. It can be affected by multiple factors such as the relative displacement between the electrode and the skin. To correctly interpret the motion intention from the measurement, possible noises must be removed. The sEMG signals from each walking trial were first band-passed with a 20–450 Hz Butterworth band-pass filter to eliminate the majority of the inherent device noise and motion artifacts. The data was then filtered through a notch filter to remove the electrical interference. In this proposed method, both filtered sEMG and knee motion modalities were segmented by a 64 ms sliding window with a half overlap increment. The length of the sliding window was set to minimize the user's awareness of the time delay of the decoder dynamical response and the classification accuracy loss for further online validation. The EMG signal starts about 20–80 ms before the muscle contraction (Copaci et al., 2018), The time that lapses between the onset of electrical activity and a measurable change in corresponding muscle tension is defined as electromechanical delay (EMD) (Cavanagh and Komi, 1979). Figure 2 provides an approximation in form of gray section masks. Due to its existence, the contralateral sEMG measured at the same time does not represent the corresponding motion of the complementary limb, and thus, the measurement mismatches the simultaneous recorded ipsilateral kinematic signals. Therefore, we manually set a phase advance shift during sEMG acquisition to compensate for the EMD. In this experiment, the EMD value for sEMG from knee extensor and flexor muscles was configured as 80 ms (Vos et al., 1991).

#### 2.1.2. Feature Extraction

We segmented the continuous trial data using a 100 ms sliding window with a half overlap for a better processing simulation which makes a performance tradeoff between the delay and accuracy. Nine features including VAR (variance), STD (standard deviation), RMS (root mean square), MAV (mean absolute value), MAX (maximum), WL (wavelength), WA (Willison amplitude), SSC (slope sign change), and iEMG (integrated EMG) were extracted from the multiple-channel sEMG signals of the dominant or unimpaired leg when sliding the window (Details of these features can be found in the Appendix). Only the mean values across the sliding windows were calculated from the knee angle and angular velocity data considering time-invariability stability during periodic walking. Then nine features per muscle (four muscles in total) and one feature from each kinematic signal (two kinematic signals in total) were fused in parallel to an *n* × *m* dimension tensor, where feature dimension *n* is equal to 38, and *m* indicated the sample points amounts of each trial.

#### 2.1.3. Labeling Four Gait Phases

In this offline mode, foot pressure captured by Zebris-FDM-T System (Zebris Medical GmbH, DE) served as the ground truth. Following the nomenclature of gait phases suggested in the article (Taborri et al., 2016), the gait cycle was divided into four phases (Figure 2): Heel Strike (HS), Flat Foot (FF), Toe Off (TO), and Swing (SW). Then motion and sEMG tensors were annotated with gait sub-phases labels.

#### 2.1.4. Architecture of Gait Phase Decoder

Given that walking is a spatial-temporal process, the gait information lies not only in each moment but also in the successive relationship. Recurrent neural networks (RNN) are the typical option in the time sequence decoding task, but a new neural network framework called Temporal Convolution Network (TCN) (Bai et al., 2018) was reported to outperform canonical RNNs such as LSTMs. Therefore, we choose the TCN as our decoder framework. Essentially, TCN is a 1D fully-convolutional network with causal convolution layers: the causal convolution represents the temporal causality, while the 1D fully-convolutional architecture with zero padding guarantees the output is of the same length as the input. Besides, modern convolution network architecture techniques, including dilated convolution and residual connections, are integrated to achieve both very deep networks and a very long effective history.

In our implementation, the input parameters include the ipsilateral knee angle and angular velocity, the contralateral sEMG from four muscles. The input size is 38 which is equal to the fusion feature set dimension. The output of TCN is the current gait phase estimation out of the four categories. We specified our network architecture with four residual blocks. Within each block, the TCN had two layers of dilated causal convolution. Each dilated causal convolutions layer had two hidden sub layers, and the dilation factor *d* increased exponentially with the depth of the network in a block (*d* = 1, 2, 4), the layer was constructed from 20 filters with the kernel size *k* of 3. Other transformations were added in succession after the basic dilated causal convolution, including weight normalization (Weight Norm), rectified linear unit (ReLU), and a spatial dropout. The inputs of the residual block were passed through the two modified dilated causal convolutions, and the outputs after these transformations were added to the inputs of the block. An illustration of one residual block can be found in Figure 1. All blocks were constructed in the same way and connected serially. After passing through four residual blocks, the results were connected to a full connection layer with zero padding, and the softmax layer determines which candidates need to be output.

#### 2.1.5. Intra-Subject Learning Strategy for the Gait Phase Classification Model

The TCN was implemented using MATLAB R2020a (The Mathworks, USA) on Lenovo ThinkStation with an Intel Xeon CPU processor E3-1225 V2 @ 3.2 GHz and 12 GB RAM, and we adopted the training strategy similar to the study by Su et al. (2020). However, another study (Nardo et al., 2020) suggested that gait event detection by neural network interpretation of intra-subject sEMG data can outperform typical inter-subject approaches. So we did not pool the data for an inter-subject test given that sEMG is highly personalized.

In the intra-subject training, we first trained the first 70% of gait data and tested the last 30% of data for each speed individually. Data was not randomized to maintain the historical sequential information. This implementation was designed to investigate whether the walking speed affects the decoding accuracy of the ipsilateral gait phases and coordination of the bilateral gait phases. Moreover, the change of the training configuration simulated the decoder's self-adaption process when the speed varies. Cross entropy is selected as the loss function, and to prevent overfitting, the classifier was trained for a maximum of 30 epochs with a 0.05 dropout factor.

#### 2.1.6. Post-processing

Post-processing is an offline analysis technique to provide a more reliable gait phase recognition performance by eliminating sporadic errors. During the model testing, we added the procedure to the abnormal pulse. If the change of the gait phase status cannot hold over a pre-defined width threshold, it can be regarded as a false estimation. This procedure is introduced to provide a smoother decoding result of gait phase transition.

#### 2.1.7. Metrics

For long-period prosthesis control, bilateral gait coordination relies on both decoding accuracy and decoding consistency. The decoding accuracy is defined as the average decoding correctness compared to the ground truth, and the decoding consistency is referred to as the variation of the decoding accuracy. The mean intra-subject classification accuracy of the decoder was calculated by comparing the predictions on the held-out test set with the true gait phase labels. Then the decoding consistency was intuitively compared *via* interquartile range (IQR) of across-speed classification accuracy of the decoder fusing either ipsilateral or contralateral sEMG. A smaller IQR indicated a better consistency.

### 2.2. Experimental Protocol

Knee motion provides abundant information about the gait phase, and the agonist and antagonist muscle groups are needed to power the joint to accomplish the walking task. Experimental evidence (Duysens and Forner-Cordero, 2018) suggests the reflex-based gait control should take hip afferents as the proprio-sensory feedback source considering its important role in loading and unloading the limb as the control input, so four coherent thigh superficial muscles were selected as the sensory feedback sources in our architecture, including Semitendinosus (ST), Rectus Femoris (RF), Vastus Medialis (VM), and Vastus Lateralis (VL). Here, the channel choice with more emphasis on the anterior is for the reason: a larger angular range of hip flexion in the sagittal plane is spotted compared to the extension during level walking, and the flexor muscles tend to stay active for a longer period.

The skin areas of interest were cleaned with 70% alcohol before data collection, and the gelled noninvasive Ag/AgCl electrodes (bipolar and diameter of 4 mm) were used to improve the contact of the electrode with the skin and obtain a reliable measurement. Following the recommendation guidance (Hermens et al., 2000), each sEMG electrode was attached to the subject's designated position with an interelectrode distance of 2cm. The electrodes were placed in parallel to the direction of the fibers to avoid the innervation zone of the muscles (Lu et al., 2019). Neural commands (sEMG from the thigh of bilateral legs in the healthy group or of the sound side in the amputee case) and the current kinematic status of knee angle and angular velocity signals from both legs were simultaneously recorded at a sampling frequency of 1,500 Hz and collected by wireless sensors through a Noraxon Direct Transmission System (DTS, Noraxon, USA). The placement of the sensors were shown in Figure 1.

Six student volunteers as the HC (healthy control) group enrolled in the experiments, and all the participants were self-reported as right leg dominant according to their handwriting habits. Besides, one unilateral HD (hip disarticulation) amputee wearing our customized lower-limb prosthesis (Li et al., 2021) enrolled in the experiments. A detailed description of all the participants is provided in Table 1. The experiments were approved by the local ethics committee and performed at the Rehabilitation Engineering Laboratory at the University of Shanghai for Science and Technology. All participants provided written informed consent prior to any procedure of the experiments. Before the experiments, subjects had enough rest to avoid muscular fatigue. Since inter-limb coordination during walking appears to be gait speed dependent (Bondi et al., 2017), we investigated the reliability of the models under different speed configurations. The healthy volunteers were asked to walk on the treadmill at their self-selected speeds and three different pre-defined speeds: slow (2.0 km/h), normal (2.5 km/h), and fast (3.0 km/h). Two trials were repeated for each speed, and each trial lasted for 60 s. Between each trial, the participants had at least 30 s rest time to relieve muscular fatigue. The amputee followed a similar instruction except the speed was only configured to his self-selected speeds (Table 1). In particular, walking training was conducted before the experiment started.

### 2.3. Statistical Analysis

The current paradigm in myoelectric control of robotic lower limb prostheses only relies on EMG from the residual limbs, a similar role of the non-dominant leg in the healthy control group, the substitution of the contralateral sEMG remains to be examined. Besides, no sEMG information can be obtained from the amputation side in the hip disarticulation amputee case, the correlation of the bilateral muscle activities can be only explored in the healthy control group. Therefore, we conducted the correlation analysis over the healthy to validate the design rationality of the algorithm fusing the bilateral information and performed the *t*-test to check whether the decoding results obtained from the bilateral information exhibit a significant difference from the ipsilateral.

Detrended cross-correlation analysis (DCCA) was applied to analyze bilateral sEMG channels because it is suitable for analyzing non-stationary time series with periodic trends (Podobnik and Stanley, 2008; Wang and Zhao, 2012). DCCA is a modification of standard covariance analysis where the global average is replaced by local trends, and its performance has been systematically tested for the effect of nonstationarities (Podobnik and Stanley, 2008; Prass and Pumi, 2021). The bilateral sEMG cross-correlation can be characterized as the *F*_DCCA_(*n*) index which is calculated in the following formula:


(1) $$\begin{array}{l} \rho_{\text{DCCA}}\left(\alpha ,\alpha^{\prime },T,n\right)=\frac{F_{\text{DCCA}}^{2}\left(n\right)}{F_{\text{DFA}}\left(n\right)F_{\text{DFA}}^{\prime }\left(n\right)} \end{array}$$

where the DFA denotes the Detrended Fluctuation Analysis which calculates the power-law auto-correlation of the ipsilateral sEMG signal, and *F*_DCCA_(*n*) and *F*_DFA_(*n*) can be defined as the following:


(2) $$\begin{array}{l} F_{\text{DCCA}}\left(n\right)\equiv \sqrt{\frac{1}{N-n}\overset{N-n}{\underset{i=1}{\sum }}f_{\text{DCCA}}^{2}\left(n,i\right)} \end{array}$$

(3) $$\begin{array}{l} F_{\text{DFA}}\left(n\right)\equiv \sqrt{\frac{1}{N-n}\overset{N-n}{\underset{i=1}{\sum }}f_{\text{DFA}}^{2}\left(n,i\right)} \end{array}$$

where *f*_DCCA(*n, i*)_ are defined as:


(4) $$\begin{array}{l} f_{\text{DCCA}}\left(n,i\right)\equiv 1/\left(n-1\right)\overset{i+n}{\underset{k=i}{\sum }}\left(R_{k}-\tilde{R_{k,i}}\right)\left(L_{k}-\tilde{L_{k,i}}\right) \end{array}$$

where the *R*_*k*_ and *L*_*k*_ are the bilateral sEMG signals, and the *f*_DFA(*n, i*)_ replaces the *R*_*k*_ and *L*_*k*_ of the ipsilateral leg in Equation 4.

In our study, R was employed to implement the DCCA. Referring to the empirical value suggested in Podobnik et al. (2011), the cross-correlations of bilateral muscle channels are considered significant if the index *F*_DCCA_(*n*) is larger than 0.185 for the 95% confidence level.

In the session of decoding accuracy analysis, a normality test was performed in the dataset with the outliers deleted by the quartile detection (threshold at 1.5). The level of significance for all statistical analyses was accepted at *p* < 0.05 unless otherwise stated. *P*-values between 0.05 *and* 0.10 were considered to indicate a statistical trend.

## 3. Results

### 3.1. Gait Phase Decoding Performance Assessment

**Figure 3:**
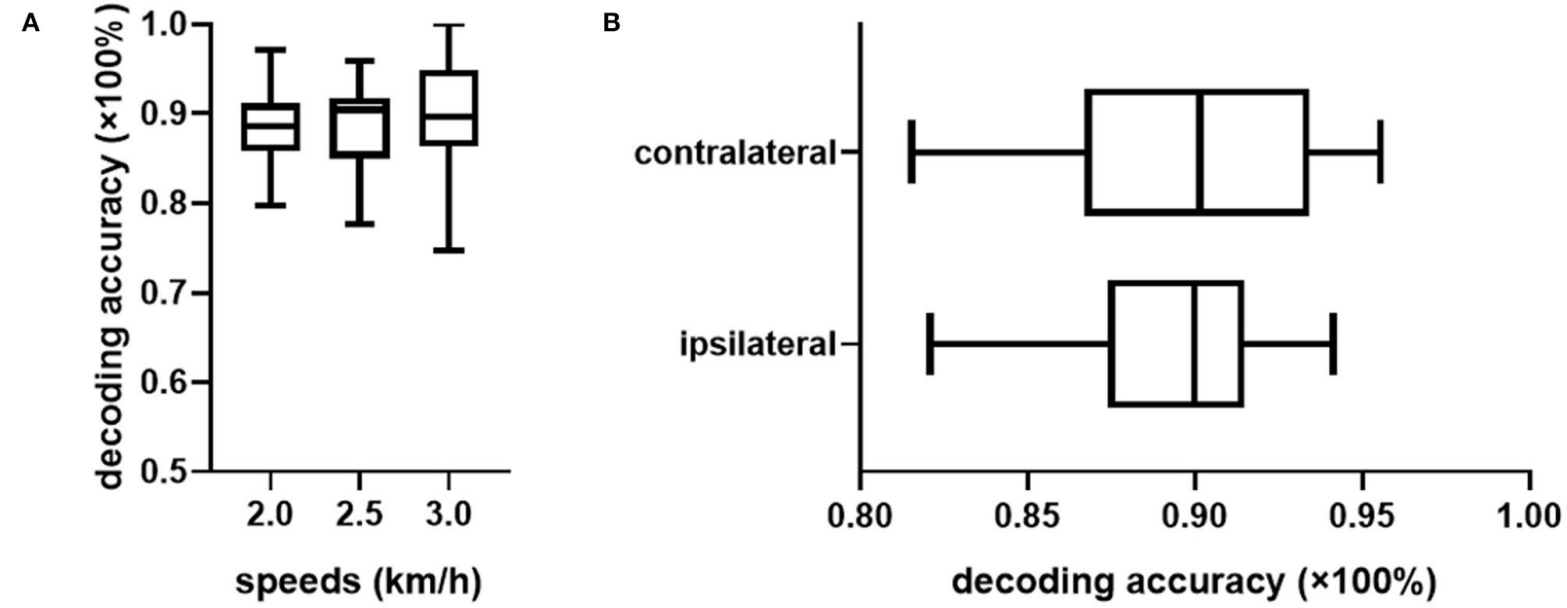
Overall evaluation of the gait phase decoder performance across the healthy subjects. **(A)** Shows the decoding accuracy under three predefined speeds. **(B)** Compares the decoding accuracy fusing sEMG sensor feedback from different legs.

The overall assessments of the decoder's ability to extract information from the ipsilateral fusion sensors using multiple-subject testing results were shown in Figure 3A. The most prominent change in the sample floating range depicted in IQR was observed at the fast speed (3.0 km/h), whereas the slow speed (2.0 km/h) remains the most stable. The measure of central tendency indicated by the medium achieved the best result (90.41%) at the normal speed (2.5 km/h). A 95% confidence interval (CI) of mean decoding accuracy was computed for each speed. The mean value at 3.0 km/h reaches the highest accuracy (91.16%), while it reaches the lowest (89.27%) at the slow speed (2.0 km/h). To check for the design rationale of fusing the contralateral sensory feedback, the result is shown in Figure 3B, where sEMG from the ipsilateral is treated as the control group. The *t*-test indicates that the gait phase decoding result exhibits no difference when we substituted the ipsilateral sEMG sensors for the contralateral as the fusion information source. Both the floating range of the decoding results and the mean value of gait phase recognition are similar. Additionally, the *p*-value (*P* = 0.98) supports the decoder's ability to handle the bilateral sensory feedback.

**Figure 4:**
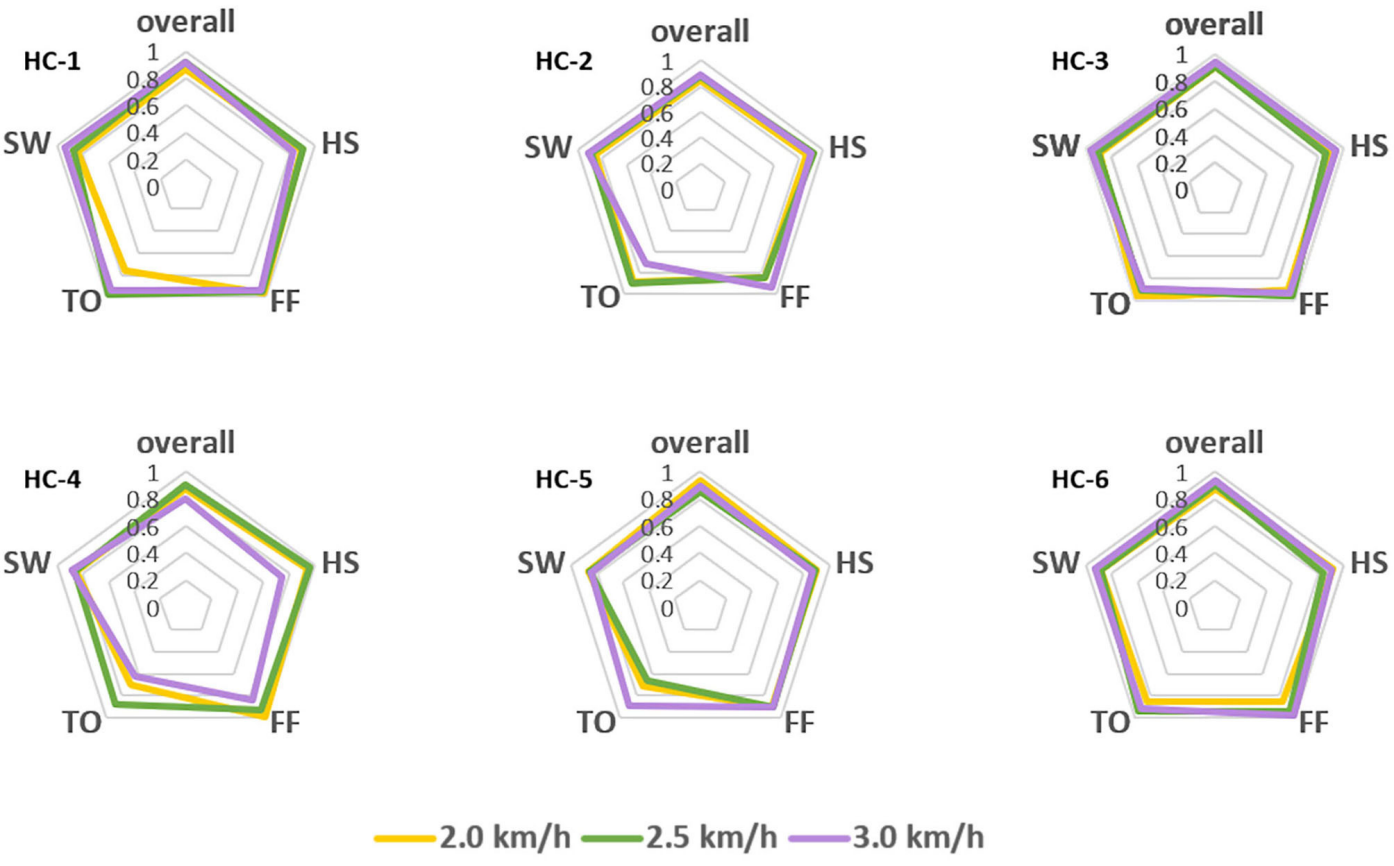
Comparison of the gait phase decoder across three predefined speeds for the six healthy subjects. Both the overall performance and the sub gait phase decoding accuracy are in the same radar chart as different axes.

**Figure 5:**
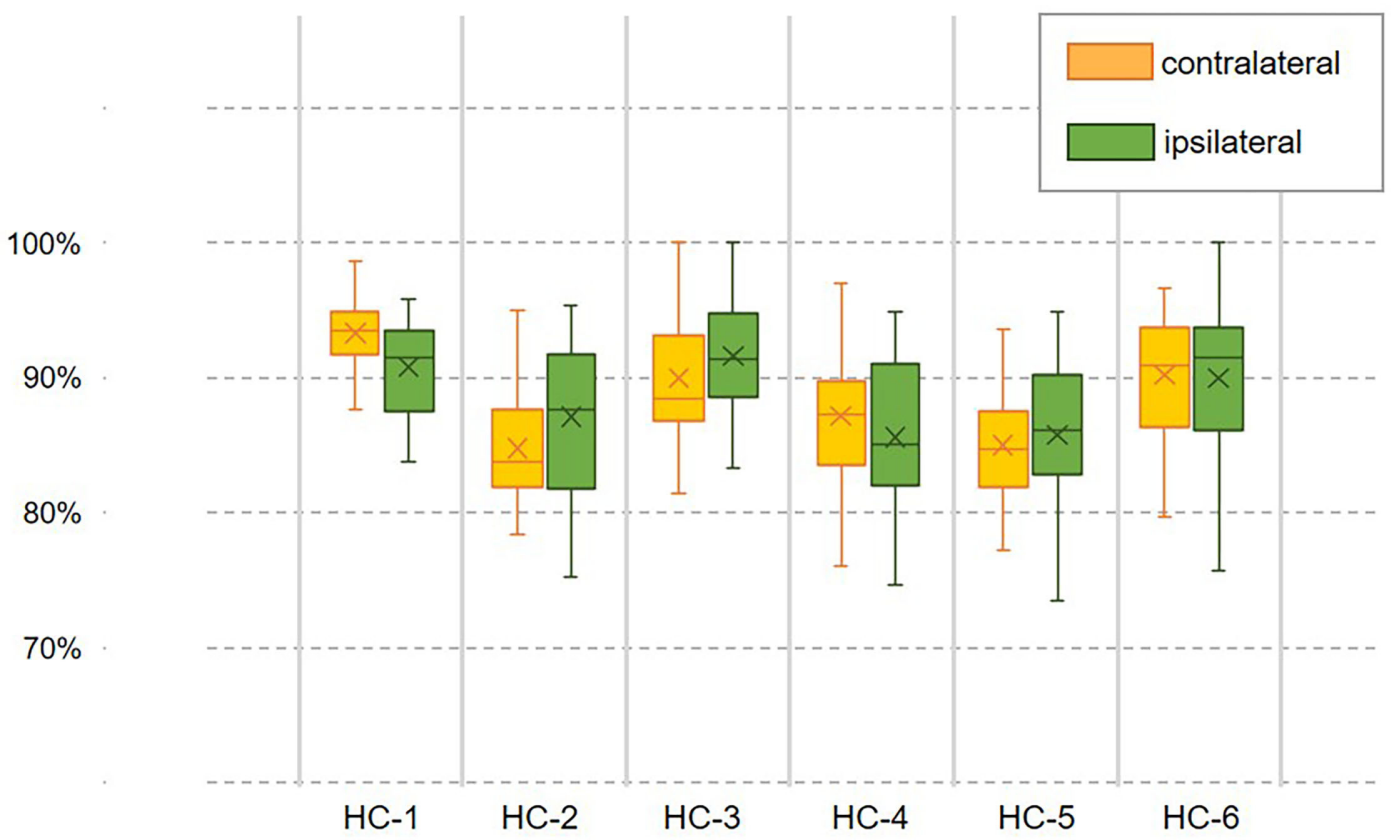
Examination of the decoder's speed adaptability when coordination information was introduced. The decoding accuracy with a fusion dataset containing the contralateral or ipsilateral sEMG is compared within the same person. The central trending indicates the decoder's consistency across speeds.

However, the pattern of muscular activities varies from person to person, the overall assessment is not complete to assess the decoder's performance, hence, we further checked the intra-subject decoding accuracy and consistency. To estimate the detailed effects of speed variability on the decoder's performance fusing ipsilateral sensors, the radar chart in Figure 4 was used to compare the overall accuracy as well as the sub gait phase recognition results across three predefined speeds for six healthy subjects, respectively. A greater covering area indicates either a higher accuracy or a more consistent decoding performance across different gait sub phases. The speed effect on the decoder varies from person to person. The HC-1, HC-3, and HC-6 reported the best decoding results as the curve covers the maximum area across speeds. Generally speaking, the maximum inference error mainly occurs at the pre-swing transition phase (TO), and the normal speed seems to be the least affected one. Speed adaptability should be considered when evaluating the decoding consistency, thus, we further mixed the multiple speed results for the same person. The central tendency described by IQR in Figure 5 indicates that the gait decoder can improve the decoding accuracy consistency for most participants when we changed the ipsilateral to the contralateral sEMG signal.

### 3.2. Cross-Correlation Analysis of Bilateral Muscle Activities

**Figure 6:**
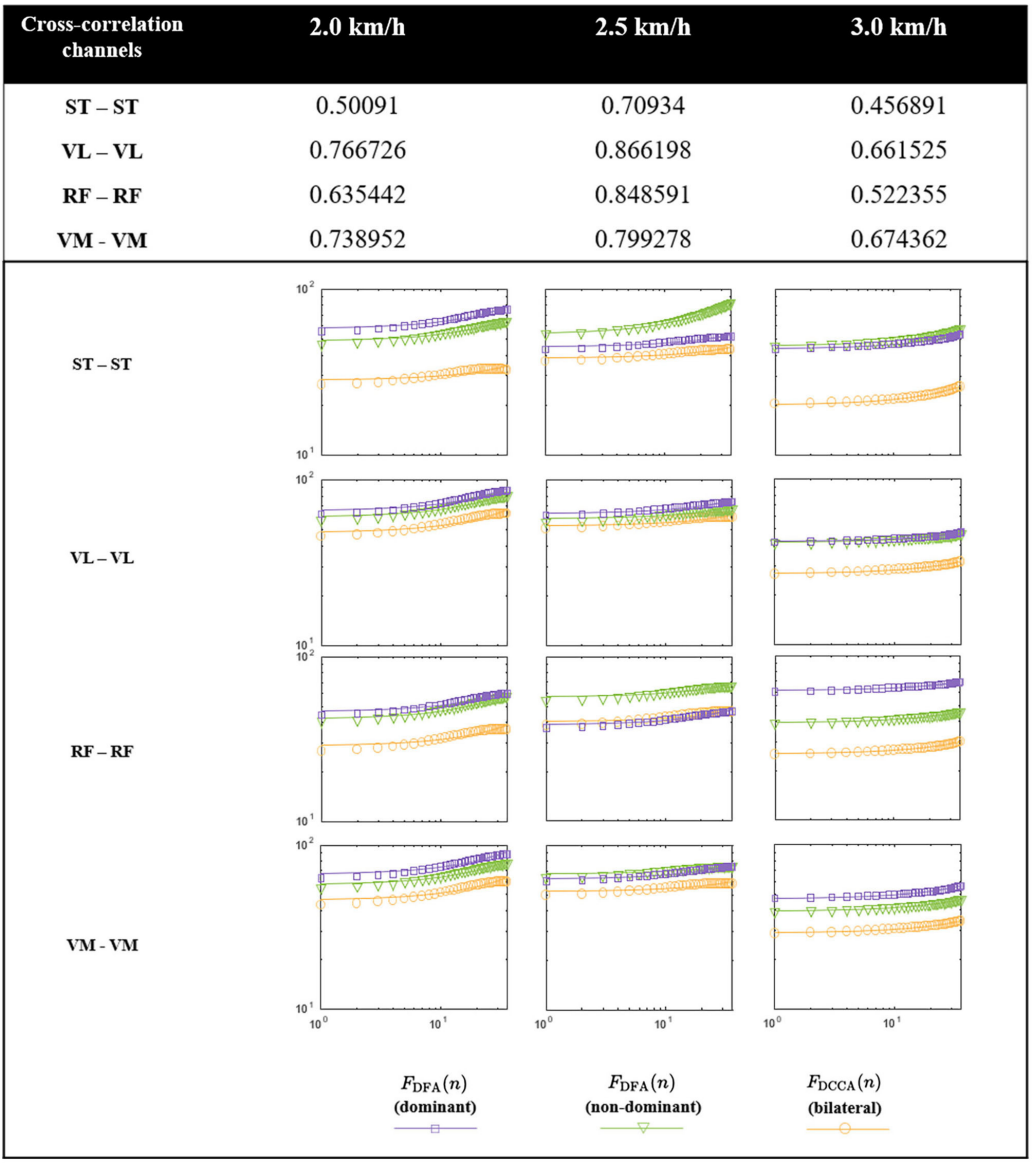
Detrended cross-correlation analysis (DCCA) results across three predefined speeds. The table above describes four muscle channels and assessment of the muscle activities contribution, and the panel below shows the cross-correlation analysis results for corresponding muscle pairs.

The results of DCCA for bilateral sEMG are presented in Figure 6. The power-law auto-correlation in DFA for sEMG of bilateral legs serves as the reference value for the DCCA. The DFA reference values for bilateral legs are almost identical across speeds and achieve the best consistency across muscles at the speed of 2.0 km/h. Among the three speed configurations, the cross-correlation of bilateral muscle activities is at a minimum gap distance from the referenced auto-correlation value at the normal speed (2.5 km/h) for all the muscle channels. The distance gap from the reference value can be quantified by the *F*_DCCA_(*n*) index, which has a physical meaning of muscle contribution in our case, varies across channels across walking speeds. Combining the DCCA index table, all channels are significantly cross-correlated, and bilateral VM-VM and VL-VL exhibit the highest relevance, then followed by RF-RF and ST-ST pairs.

### 3.3. Validation in Amputee Gait Phase Estimation

**Figure 7:**
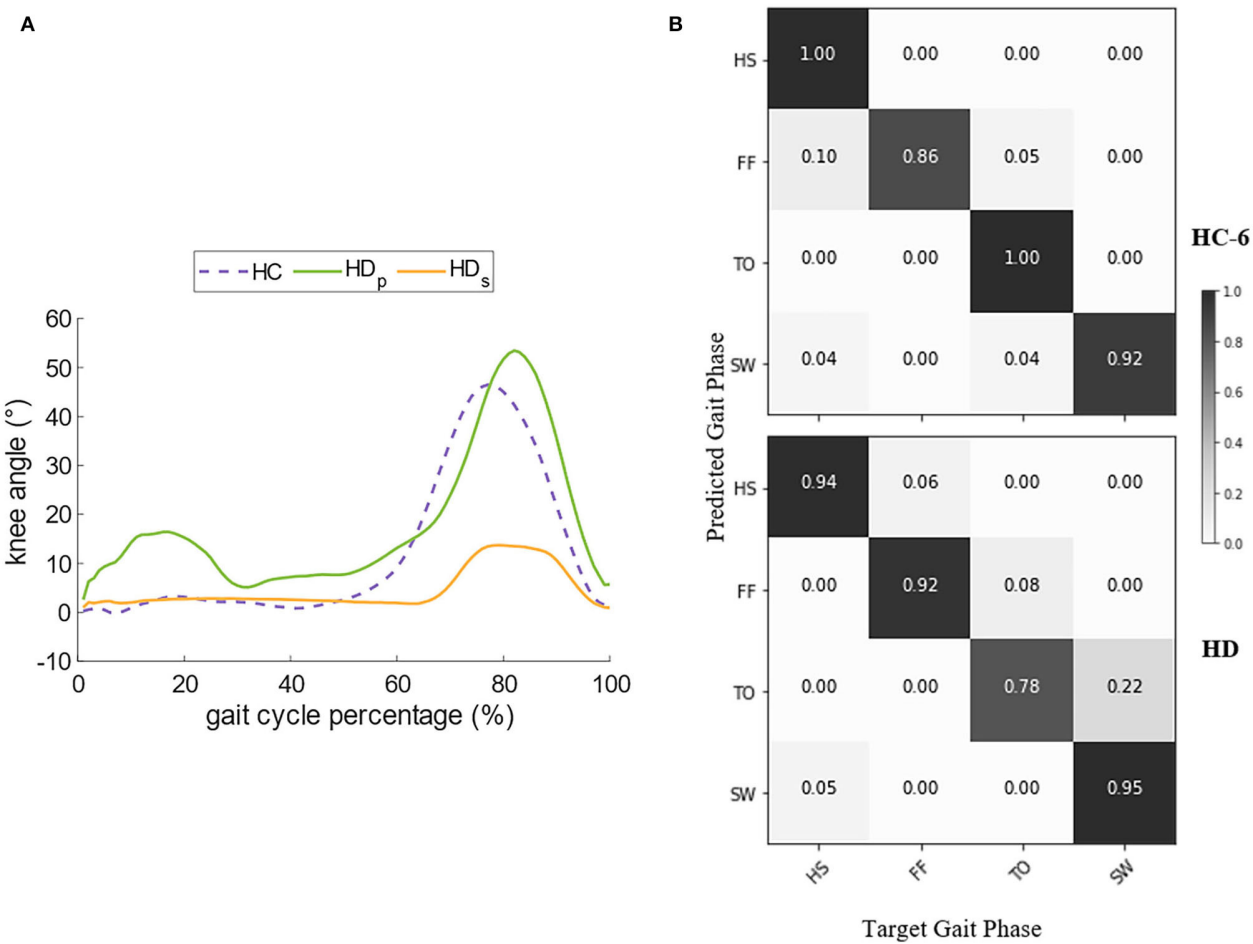
Comparison of the knee angle and the decoder's performance between HC (Healthy Controls) and HD (Hip Disarticulation) groups. **(A)** Compares the dominant knee angle of HC and the bilateral knee angle of HD, and **(B)** compares the decoder's performance in the confusion matrix.

As walking speed is a prominent factor in gait pattern generation, we chose the record of Subject HC-6 for further comparison since his self-selected speed is the same as the HD's (Table 1). Figure 7A compares the angles of knee flexion and extension between the hip disarticulation amputee and one healthy subject. Generally speaking, the amplitude and the sub gait phase duration of sound-side knee angle for the amputee is very similar to the healthy, whereas the angle amplitude of the prosthetic side is much smaller and the stance phase tends to last longer. The confusion matrix in Figure 7B compares the gait phase decoding results between the two groups. At the self-selected speed, though the overall gait phase decoding accuracy shows a significant difference (*P* = 0.0036) between groups, both groups reported a high accuracy (90.30% for the amputee and 93.00% for the subject HC-6). In both cases, the errors mainly occur during the continuous transition of the gait phase. Besides, there is a greater probability for the decoder to make the wrong estimation about the gait phase of the prosthetic side during the stance phase compared to the healthy control group.

**Table 1:** Personal information of participants.

**Participant ID**	**Sex (m/f)**	**Age (year)**	**Height (m)**	**Weight (kg)**	**Self-selected speed (km/h)**	**Dominant/Unimpaired lateral (L/R)**
HC-1	f	24	1.70	48	2.80	R
HC-2	f	24	1.57	50	2.70	R
HC-3	f	27	1.68	55	2.90	R
HC-4	f	22	1.55	53	2.50	R
HC-5	m	23	1.70	65	2.50	R
HC-6	m	23	1.72	72	1.80	R
HD	m	32	1.75	60	1.80	L

## 4. Discussion

### 4.1. Biological Implications for Movement Pattern Generation

One hypothesis about the architecture of MPG involves a Central Pattern Generator (CPG) and a neural reflex loop (Dzeladini et al., 2017). The CPG functions as the endogenous oscillator to generate the gait pattern with appropriate phase delays (Figure 2), and the reflex loop perceives the state of the body in a certain environment from the sensory feedback. In our architecture, the TCN can model the gait phase delays in a black-box paradigm. As a result, by continuously fusing the bilateral information, it can model the merged behaviors of the CPG and the reflex loop.

The formation of MPG can be considered as entrainment of the cyclic motion of bilateral legs with response to internal motion intention commands and adaptation to external environment disturbance. Furthermore, the coupling process modeled by our architecture can be interpreted as intra-system communication and inter-systems communication (Dzeladini et al., 2017). The former guarantees gait coordination during the pace transition, while the latter guarantees a high efficiency human-to-robot interaction. This makes it especially suitable for enhancing the safety in the complex amputee-prosthesis-system since the gait coordination errors are the compounding results of time-lag response in gait pattern transition as well as a deficiency of the communication inefficiency between the biological system and the physical system. Besides, by utilizing the physiological sensory feedback to decode the motion intention of the amputee subsystem, the decoder provides voluntary control over the contralateral artificial leg and allows for a faster response to achieve inter-leg coordination.

### 4.2. Explanation of Speed Effects on Decoding Accuracy

Gait phase decoding accuracy is the first step toward inter-leg coordination. Overall performance of the decoder was consistent across speeds for most subjects (Figure 7), this implies that the gait phase decoder was robust to the change of the gait speeds, and this property corresponds to the MPG's robustness to perturbations during walking. Additionally, the variation of speeds mainly takes effect at the transition between the swing and stance phase, this may be the misinterpretation of sensory feedback when the decoder fuses the contralateral sEMG to make a judgment. At different speeds, one possible reason for the difference in contribution index (Figure 6) is that the muscle activities tend to last longer and the recruitment amount of muscle fibers is larger at a slower speed, so the nonlinearity occurs with a higher probability, and this leads to the loss of accuracy. However, this may no longer hold true taking into account the slower self-selected pace. In these cases, muscles contract with regularity to minimize the energy cost.

When comparing the same speed between the healthy control group and the amputee, a decoding accuracy loss was spotted in the amputee's case. The decoder makes the maximum errors when it infers the amputee's gait phase at the TO and mistakes it as the FF. This can be partly explained as the knee joint trajectory of the prosthetic leg shows a smaller amplitude compared to the healthy leg (Figure 7A). The smaller displacement indicates a more stiff status of the prosthetic leg, and thus, the sub gait phases are not so distinctive. The walking habit may partly account for it. Considering that the amputee may still not get used to the prosthetic leg even after a period of walking training before the start of the experiment, the neural system may generate an irregular motor pattern to prevent fall risks, and thus, the level of muscle activities tends to be stronger (Yang et al., 2007) to coordinate the sound side, as a consequence, a great exposure chance for sEMG exhibiting the time-variant property can result in the decline of recognition accuracy.

Although our architecture has followed the sensor fusion suggestion in literature (Hamzaid et al., 2020), and the statistical analysis results (Figures 3B, 6) indicated a high bilateral correlation and spotted no significant decoding difference when the modality choice changed, the time-variant sEMG can still confuse the decoder from time to time; this calls for the further investigation in improving the motion intention estimation accuracy.

### 4.3. Comparison to the Previous Studies

The proposed architecture shares similar inspiration from nature with the artificial CPG implemented widely in the legged robots (Ryu et al., 2009; Akkawutvanich et al., 2020; Tanikawa et al., 2021), but the latter only provides the trajectory control solution, while our design can provide a wider choice for the low-level controller with additional control hierarchy adding in. Echo control is a similar implementation in the field of lower-limb prosthesis control, however, it is still based on the trajectory control paradigm. A gait phase decoder based on echo control can be found in Wang et al. (2013), but it lacks the perception of the human motion intention from the internal sensory feedback which is critical in the human-centered design.

Limited research has investigated the utilization of bilateral modalities which contain meaningful interlimb coordination information, such as the gait phase delay across the legs, not to mention fusing the contralateral neural information to additionally extract the motion intention. The decoding accuracy exhibits no difference when we replaced the ipsilateral sEMG with the contralateral sEMG (Figure 3B) with a *p*-value indicator of 0.98, this supports the complementary myocontrol rationale with respect to myocontrol from the residual limb (Fleming et al., 2021). Moreover, as indicated in Hu's research (Hu et al., 2018), bilateral sensors fusion can reduce steady-state and transitional error rates in locomotion recognition. The distinction may be a result of bilateral sensor amounts used in a different recognition task. Incorporating signals from both legs also allows the gait phase decoder to make a decision with interlimb coordination information. The deviation of the bilateral decoding results in Figure 5 indicates a distinctive reduction of the fluctuation compared to the unilateral in most cases. A potential explanation is that fusing the bilateral gait phase will reduce temporal uncertainty in the decoder's inference. Upcoming gait phase estimation is relevant to not only the current ipsilateral gait phase but also the gait phase of the contralateral leg. Missing the contralateral gait information may lead to a "blind" inference which further causes an inappropriate dynamic response while increasing the risk of falling. Besides, by incorporating the contralateral sEMG, an internal lower-limb motion status of the participant can be obtained to constrain the decoder's inference to improve the decoding consistency. This improved decoding reliability is critical for the design of the control system of the lower-limb walking assistance devices for safety.

### 4.4. Potential Applications and Future Developments

The proposed decoder is intended for the prosthetic leg design with an emphasis on hip disarticulation. Its application is illustrated in Figure 1 where the decoder serves as a high-level control input. By mapping the control constraints into the sub-gait phase, a finite-state-machine-like control schema can be adopted. Generally speaking, there should be a more strict control constrain of the prosthetic leg during the bodyweight supporting phase for safety considerations, especially in complex locomotion environments. The proposed decoder shows the promise of a smoother transition during the stance phase. With a further improvement in accuracy, the proposed decoder can be adopted in other walking assistive devices design, such as lower-limb exoskeletons and prostheses.

As indicated in Englehart and Hudgins (2003), a control system must respond within 300 ms so that the user will not perceive the delay, and for the control system design in the lower-limb application, this threshold should be smaller to guarantee the safety of the user. Offline evaluation of this decoder shows the total time per step is about 2 ms using a 64-ms time window on the aforementioned hardware. For an embedded control system, the computation time should be further reduced with model optimization. Another limitation of this study is that we only consider the subdivision of the stance phase. The swing phase with a finer granularity should be further considered in the prosthetic leg control system design.

## 5. Conclusion

In this study, we have addressed the gait phase decoding problem inspired by the hypothesized MPG architecture to achieve gait coordination in the amputee-prosthesis coupling system. TCN has been employed to model the behaviors of gait pattern generator across different speeds, and sensory feedback from bilateral legs has been fed into the TCN where the sEMG has been utilized to estimate the human's motion intention. The decoder has exhibited a high intra-subject consistency in the gait phase inference, adapted to various paces with a tolerant decoding accuracy loss, and it has achieved a high accuracy in sub phase estimation of the prosthetic leg in the hip disarticulation amputee wearing our customized prosthesis. These results have suggested a possible improvement in walking coordination with an intelligent prosthesis for the amputees at hip level amputation. Further study will integrate the gait phase decoder in the control schema of the intelligent prosthesis and validate it in the online mode.

## Data Availability Statement

The raw data supporting the conclusions of this article will be made available by the authors, without undue reservation.

## Ethics Statement

The studies involving human participants were reviewed and approved by the Local Ethics Committee at Rehabilitation Engineering Laboratory in the University of Shanghai for Science and Technology. The patients/participants provided their written informed consent to participate in this study.

## Author Contributions

YC, XL, and HY conceptualized and designed the study. YC measured and analyzed the participant's data in consultation with XL. YC drafted the manuscript. XL, HS, and DZ revised the article critically for important intellectual content. All authors contributed to the article and approved the submitted version.

## Funding

This study was supported by a grant from the National Key Research and Development Program of China (no. 2018YFB1307301).

## Conflict of Interest

The authors declare that the research was conducted in the absence of any commercial or financial relationships that could be construed as a potential conflict of interest.

## Publisher's Note

All claims expressed in this article are solely those of the authors and do not necessarily represent those of their affiliated organizations, or those of the publisher, the editors and the reviewers. Any product that may be evaluated in this article, or claim that may be made by its manufacturer, is not guaranteed or endorsed by the publisher.

## Appendix

The math equations and symbols of the feature extractors are listed as the following:

VAR (variance):

$$VAR=\frac{1}{N-1}\overset{N}{\underset{i=1}{\sum }}{\left(X_{i}-\overset{-}{X}\right)}^{2}$$

STD (standard deviation):

$$STD=\sqrt{\frac{1}{N}\overset{N}{\underset{i=1}{\sum }}{\left(X_{i}-\overset{-}{X}\right)}^{2}}$$

RMS (root mean square):

$$RMS=\sqrt{\frac{1}{N}\overset{N}{\underset{i=1}{\sum }}{\left(X_{i}\right)}^{2}}$$

MAV (mean absolute value):

$$MAV=\frac{1}{N}\overset{N}{\underset{i=1}{\sum }}|X_{i}|$$

MAX (maximum):

$$MAX=maximum\;\left(X_{i}\right)\;where,i=1,...,N$$

WL (wavelength):

$$\text{WL}=\overset{N-1}{\underset{i=1}{\sum }}|X_{i+1}-X_{i}|$$

WA (Willison amplitude):

$$\begin{array}{c} \text{WA}=\overset{N}{\underset{i=1}{\sum }}f|X_{i}-X_{i+1}|\\ f\left(X_{i}\right)=\{\begin{array}{c} 1\&\text{if }X>\text{thresholding}\\ 0\&\text{otherwise} \end{array} \end{array}$$

SSC (slope sign change):

$$\begin{array}{c} \text{SSC}=\overset{N-1}{\underset{i=2}{\sum }}f\left(X_{i}\right)\\ f\left(X_{i}\right)=\{\begin{array}{c} 1,\text{if }\left\{\left(X_{i}>X_{i-1}\&X_{i}>X_{i+1}\right)|\left(X_{i}<X_{i-1}\&X_{i}<X_{i-1}\right)\right\}\\ \&\left\{\left(|X_{i}-X_{i+1}|\geq T\right)|\left(|X_{i}-X_{i-1}|\geq T\right)\right\}\\ 0,\text{otherwise} \end{array} \end{array}$$

iEMG (integrated EMG):

$$\text{iEMG}=\int_{t}^{t+N}|X_{i}|\text{d}t$$
